# Repeated CD107a Staining Enables Identification of Serial Degranulating NK Cells

**DOI:** 10.1002/eji.202451642

**Published:** 2025-05-09

**Authors:** Jens Niemann, Maren Claus, Vanna Imširović, Carsten Watzl

**Affiliations:** ^1^ Department for Immunology Leibniz Research Centre for Working Environment and Human Factors (IfADo) Dortmund Germany; ^2^ Department of Histology and Embryology Faculty of Medicine University of Rijeka Rijeka Croatia

**Keywords:** cytotoxicity, Innate lymphocytes, natural killer cells, serial killing

## Abstract

Upon repeated target cell contact serial degranulating NK cells are identified by multiple staining events using differentially labeled anti‐CD107a (LAMP1) antibodies. This flow‐cytometry‐based method allows for the characterization and isolation of serial degranulating NK cells.

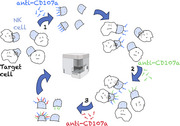

Natural killer (NK) cells are important innate lymphocytes for early and effective immune responses against transformed or virally infected cells [1]. NK cell cytotoxicity is executed by the release of cytotoxic granules or the engagement of death receptors [2]. Human NK cells have been shown to be heterogeneous and differ in their ability to kill target cells [3]. NK cells that can sequentially kill multiple target cells are called serial killers [4]. The serial killing activity of NK cells is essential for their cytotoxic function since the majority of kills can be performed by a minority of cells [5]. Consequently, serial‐killing NK cells are of particular interest for therapeutic applications [6]. Mechanistically, we could previously show that serial killing NK cells use granzyme and perforin‐mediated cytotoxicity for their first kills while they switch to death‐receptor‐mediated apoptosis for their final kill [7]. However, while it is known that only a fraction of NK cells perform serial killing, it is currently not possible to predict which NK cells will engage in serial killing. Many existing methods utilize microscopy to identify serial killing NK cells, which is low in throughput and does not allow for easy characterization of serial killers. Therefore, we wanted to use flow cytometry to identify and better characterize serial‐killing NK cells.

CD107a is a commonly used degranulation marker [8] which is externalized upon granzyme/perforin and death receptor‐mediated cytotoxicity [7]. We use serial degranulating NK cells as a proxy for serial killers and identify serial degranulating NK cells by sequential staining with differentially labeled anti‐CD107a antibodies during target cell co‐culture. Once externalized by degranulating NK cells, CD107a was detectable on the cell surface for up to one hour before decreasing by internalization (Figure S1A,B). To enable the identification of repeated degranulation events, we first tested the abundance of unbound (antibody accessible) CD107a before or after staining using a fluorescently labeled anti‐CD107a antibody, or after blocking with excess nonconjugated anti‐CD107a antibody (Figure S1C). This led us to include a blocking step into the protocol, that significantly reduced the amount of CD107a that could still be detected on the NK cell surface without interfering with the initial staining (Figure S1D,E). Our optimized staining protocol (Figure 1A and supporting information for detailed methods and protocol) can detect up to three different degranulation events. PBMCs and K562 target cells are mixed and spun down enabling contact formation. To ensure proper conditions for NK cells, samples are kept at 37°C and 5 % CO_2_ during the entire degranulation and staining process. After 10 min of co‐incubation, the first anti‐CD107a antibody is added, and cells are mixed and resuspended to disrupt contacts. After 5 min blocking antibody is added to bind all available CD107a, and cells are resuspended again. After another 5 min cells are washed, resuspended in fresh media, and directly spun down again to enable new contact formation for another 10 min. For the second CD107a staining the process is repeated. The staining step is carried out with the same clone and amount of anti‐CD107a antibody but linked to a different fluorophore. After the third spin down and incubation time, cells are stained with a third fluorophore‐linked anti‐CD107a‐antibody for 5 min. After washing, cells are put on ice for phenotype staining, followed by fixation and measurement by flow cytometry. We opted for short co‐incubation times with quick CD107a staining periods to prevent loss of antibody staining or exchange of antibodies over time and to only identify fast and strong degranulating NK cells.

**FIGURE 1:**
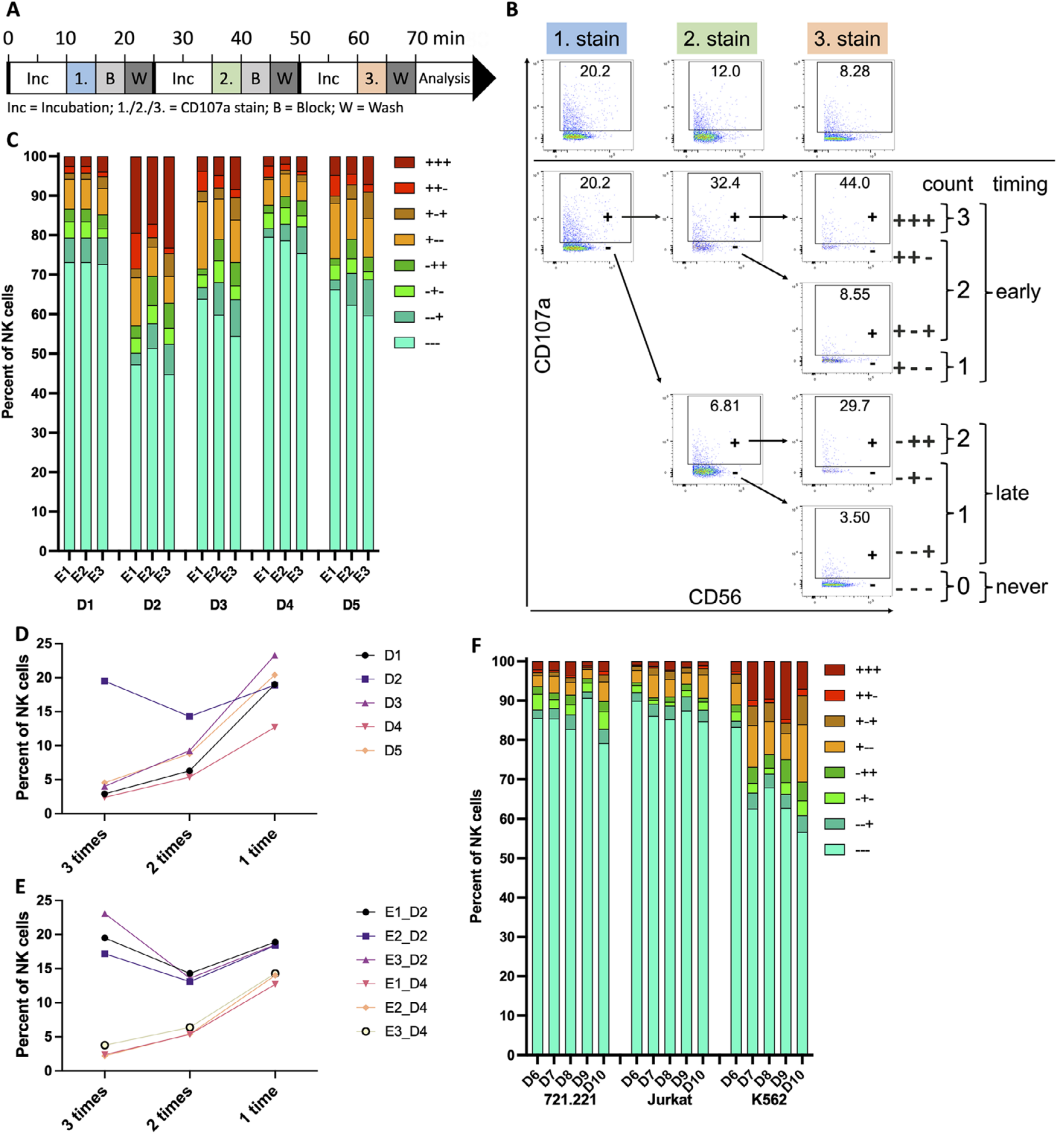
Biological variance outweighs interexperimental variance. (A) Timeline of multiple degranulation protocol, see text and supporting information for details. (B) Example of the gating strategy of a multiple degranulation assay of PMBCs and K562 targets at a ratio of 1:1. Shown are CD107a positive NK cells from the three CD107a stainings of total NK cells (top, gated according to Figure S3) or nested gating according to decision‐tree (bottom) for eight different degranulation outcomes. (C) Degranulation outcomes of a multiple degranulation assay using thawed PBMCs from five donors (D1–D5) at three independent time points (E1–E3) with K562 as target cells at a ratio of 1:1. (D, E) Percentage of NK cells having degranulated thrice, twice or once for donors and repeats from (B). (F) An assay was performed with 721.221, Jurkat, or K562 target cell lines using PBMCs from five donors (D6–10) at a ratio of 1:1.

The staining with three differentially labeled CD107a antibodies results in eight different possible staining patterns (Figure 1B). These can be condensed according to the total number of degranulation events (0–3) or according to the timing of the degranulation events (“early”, cells that degranulated during the first incubation period; “late”, cells that did degranulate but not during the first incubation period; “never”, cells that did not degranulate) (Figure 1B). Using PBMCs from five healthy donors (D1–D5) and K562 as target cells our assay revealed differences in serial degranulation patterns with one individual having up to 20% of triple degranulating NK cells while others having less than 5% (Figure 1C,D). To validate interexperimental consistency, we performed the assay on three separate days (E1–E3) using PBMCs from the same five donors (D1–D5). The assay showed very similar degranulation patterns between repeats, proving interexperimental consistency (Figure 1C,E; Figure S2). Next, we tested how different target cells influence the serial degranulation behavior of the NK cells and compared 721.221, Jurkat, or K562 cells using PBMCs from five healthy donors (D6–D10) as effector cells. We observed the largest number of serial degranulating NK cells using K562 targets consistent with the fact that these cells most effectively stimulate NK cell activities (Figure 1F). However, degranulation events using 721.221 or Jurkat targets were also specific, as we did not observe significant CD107a staining in the absence of target cells (Figure S2D). Therefore, our assay can be performed using different target cells depending on the research question.

We used PBMCs for our assay to avoid unnecessary manipulation of the NK cells. Comparing PBMCs with isolated NK cells from the same donor resulted in very comparable results (Figure S2E), demonstrating that NK cell isolation is not necessary to detect reliable NK cell degranulation using our assay. In contrast, using feeder cell expanded, preactivated NK cells resulted in more than 60% triple degranulating NK cells with little donor‐to‐donor variability (Figure S2E). Therefore, our assay can be used to easily identify donors with large amounts of serial degranulating NK cells and to test different stimulation protocols for their effect on the serial degranulation activity of NK cells.

To further test our assay setup, we additionally stained for the activation marker CD69 and the Fc receptor CD16. CD69 staining differed between degranulation groups with higher expression only in groups that degranulated during the first staining cycle (Figure 2A,B). Due to the short timeframe of our assay, only cells degranulating, in the beginning, may have enough time to upregulate CD69. In line with this, we observed CD69 upregulation also on late degranulating NK cells when increasing the incubation times (data not shown). Therefore, degranulation correlates with CD69 upregulation in our assay. CD16 is activation‐dependently cleaved from the NK cell surface [9, 10]. CD16 staining differed between degranulation groups and NK cells that did not degranulate showed the highest expression levels, while CD16 staining levels were lower for each additional degranulation event (Figure 2C,D). This confirms that the degranulation events we detect correlate with NK cell activation and that the triple‐degranulating NK cells have been stimulated by repeated target cell contact.

**FIGURE 2:**
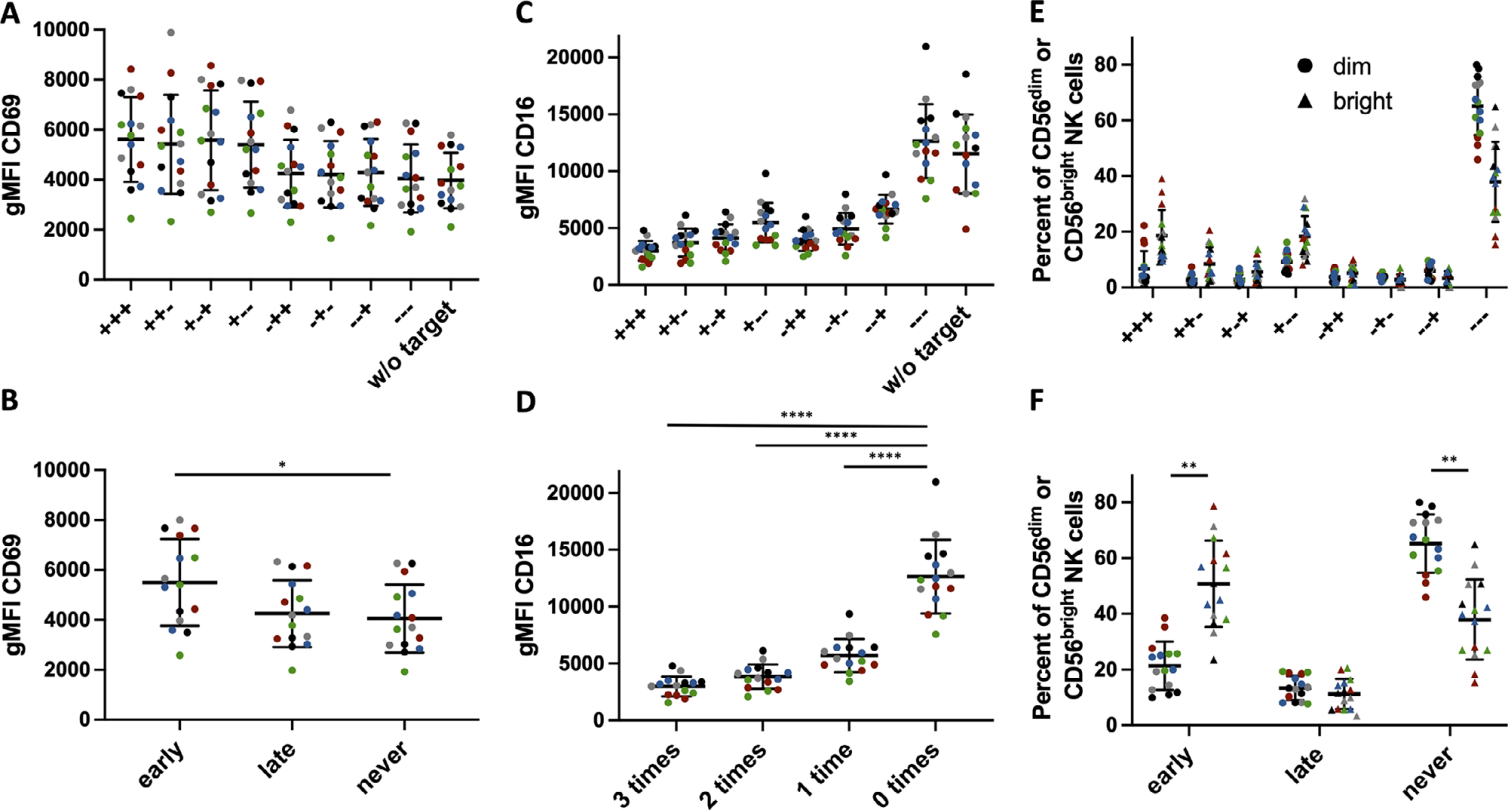
Degranulation outcome can predict CD69 and CD16 expression while CD56 expression predicts degranulation timing. Multiple degranulation assay of PBMCs and K562 targets at a ratio of 1:1 (A, B) CD69 expression on NK cells after different degranulation outcomes (A) or grouped according to degranulation timing (B). (C, D) CD16 expression on CD56^dim^ NK cells after different degranulation outcomes (C) or grouped according to the number of degranulation events (D). (E, F) Distribution of CD56^dim^ and CD56^bright^ NK cells among different degranulation groups (E) or grouped according to degranulation timing (F). Mean and SD. Nested 1 way ANOVA with multiple comparison *p ≤ 0.05, **p ≤ 0.01, ***p ≤ 0.001, ****p ≤ 0.0001. *n* = 15 (three repeats of five different donors (marked by different colors) as in Figure 1C).

Since our approach is flow cytometry based it can easily be coupled to downstream phenotyping. We used CD56 to identify NK cells among PBMCs, enabling us to distinguish CD56^bright^ and CD56^dim^ NK cells. Interestingly, CD56^bright^ NK cells degranulated faster and had a larger proportion of triple degranulating cells compared with CD56^dim^ NK cells (Figure 2E,F), which also correlated with a trend toward higher CD69 expression (Figure S2F), consistent with the findings of others [11]. However, as the more cytotoxic CD56^dim^ NK cells represent 90% of NK cells in PBMCs [12], they also make up the majority of triple‐degranulating NK cells (Figure S2G).

Our assay will be useful to identify, characterize, and isolate serial degranulating NK cells, which will enable testing these cells for their serial killing activities. This may improve stimulation methods and cell sources for using NK cells in therapeutic applications.

## Conflicts of Interest

The authors declare no conflicts of interest.

### Peer Review

The peer review history for this article is available at https://publons.com/publon/10.1002/eji.202451642.

## Supporting information



Supporting information

## Data Availability

The data that support the findings of this study are available from the corresponding author upon reasonable request.
