# Supplementary material for: Repeated CD107a Staining Enables Identification of Serial Degranulating NK Cells
Source: Eur J Immunol. 2025 May 9;55(5):e202451642. doi: 10.1002/eji.202451642 (PMC12064871; doi:10.1002/eji.202451642)
Supplement: Supplementary file 1 — Supporting information [file EJI-55-e202451642-s001.pdf]

Supporting Information

Repeated CD107a staining enables identification of serial degranulating NK cells

Jens Niemann[1], Maren Claus[1], Vanna Imširović[2], Carsten Watzl[1]

[1] Department for Immunology, Leibniz Research Centre for Working Environment and Human Factors (IfADo), Dortmund, Germany.
[2] Department of Histology and Embryology, Faculty of Medicine, University of Rijeka, Rijeka, Croatia.

Methods

Cell culture

K562 cells were cultured in IMDM (10% FCS, 1% penicillin/streptavidin) in T75 culturing flasks at 37 °C and 5% CO_2. Cells culture was split three times per week. For experiments K562 cell culture was split the day before to a concentration of 3 x 10^5 cells/mL. Jurkat cells were cultured in RPMI (10% FCS, 1% penicillin/streptavidin) in T75 culturing flasks at 37 °C and 5% CO_2. Cells culture was split three times per week. For experiments Jurkat cell culture was split the day before to a concentration of 3 x 10^5 cells/mL. 721.221 cells were cultured in IMDM (10% FCS, 1% penicillin/streptavidin) in T75 culturing flasks at 37 °C and 5% CO_2. Cells culture was split three times per week. For experiments 721.221 cell culture was split the day before to a concentration of 3 x 10^5 cells/mL. HeLa-CD48 cells were cultivated in DMEM (10% FCS, 1% penicillin/streptavidin) supplemented with 1 µg/mL Puromycin for CD48 selection in T75 culturing flasks at 37 °C and 5% CO_2. TrypLE^{TM} Express^{TM} was used to detach the cells for splitting. Cell culture was split three times per week according to their confluence.

PBMC isolation

Peripheral blood mononuclear cells (PBMC) were isolated from fresh blood of healthy donors by density centrifugation. Lymphocyte Separation Medium (LSM) was covered by a layer of blood and centrifuged (1025 x g for 25 min). The white layer containing the PBMCs was harvested. PBMC were used directly for NK cell isolation or frozen in FCS with 10 % DMSO for later use.

Preactivated NK cell culture

Fresh NK cells were isolated from PBMCs using the *Dynabeads® UntouchedTM Human NK Cells Kit* (Thermo Fisher) following manufacturer's instructions. NK cells were stimulated with _K562-mbIL15-mbIL21-41BBL and 200 U/mL IL-2. They were cultured for at least 3 weeks before experiments in IMDM GlutaMAX^{TM}(10% FCS, 1% penicillin/streptavidin) with 100 U/mL IL-2

CD107a surface retainment

HeLa-CD48 cells were seeded 0.5 mio/well in 6 well plates and allowed to adhere over night. The next day 0.5 mio/well preactivated NK cells were added, and 60 min later NK containing supernatant was removed, and remaining NK cells washed off with PBS twice. Supernatant and washed off NK cells were combined. Cells were spun down, supernatant discarded, and NK cells resuspended in fresh media. Cells were gently tumbled in the incubator to inhibit reattachment in case of carry over HeLa cells. Anti-CD107a-FITC antibody (see table 1) was added at indicated timepoints for 5 minutes, cells were washed and then fixed for 10 minutes with 2% PFA.

CD107a blocking

K562 cells and preactivated NK cells were co-incubated at an E:T of 1:2 for 40 minutes. Either BV421 conjugated anti-CD107a antibody, 10-fold unconjugated anti-CD107a antibody (see table 1) or nothing was added for 5 min. Cells where washed and directly treated with FITC conjugated anti-CD107a antibody (see table 1) for 5 minutes. Afterwards cells were washed

again, fixed for 10 minutes with 2% PFA, stained with CD56 PE (see table 1) in PBS + 2% FCS for 15 minutes, washed and analyzed by flow cytometry.

Testing CD107a blocking during multiple degranulation assay

Multiple degranulation assay was carried out as described in *Methods: Multiple degranulation assay* (below) until the second CD107a staining. Second blocking and third CD107a staining were skipped and directly continued with the dead cell staining. From there the protocol was followed to the end. The first blocking step was either done as described or without antibody.

Multiple degranulation assay

PBMCs were thawed the day before the experiment and cultured overnight in IMDM GlutaMAX™(10% FCS, 1% penicillin/streptavidin) with the addition of 0.5 ng/mL IL15 at 37°C and 5% CO_2. Freshly isolated NK cells were rested overnight after isolation in the same manner. The assay was performed in 96-well-v-bottom-plates, this enables manipulation of 12 samples in parallel in one row. 1 million K562 cells (,721.221 cells or Jurkat cells in case of Figure 1F) were used as target per well. 1 million PBMCs (0.1 million fresh or preactivated NK cells in case of Supplementary Figure 2E) were used as effector cells to gain an E:T ratio of roughly 1:10. To keep the pH constant even while handling the cells, the assay was performed in IMDM (10% FCS, 1% penicillin/streptavidin) + 10 mM HEPES (assay medium). 10 x predilutions of the anti-CD107a-antibodys (see table 1) were prepared beforehand in assay medium and pre-placed in individual rows of another 96-well-v-bottom-plate. 2 x predilution (2 x master mix) of phenotyping antibodies (CD3, CD56, CD16, CD69; see table 1) is prepared in PBS + 2% FCS. This plate is kept in the dark and on ice. Use of multi-channel pipets to handle the samples in parallel is advised.

Procedure:
-Add effector cells to V-bottom plate, spin down (5 minutes at 500 g) and remove supernatant.
-Add 50 µL assay medium with target cells or without (no target control) to the effector cell pellet.
-Resuspend and mix cells by pipetting
-Spin down for 10 seconds to enable direct contact formation
-Incubate the plate for 10 minutes at 37 °C and 5% CO_2
-Add 5 µL 10x anti-CD107a-BV421 and resuspend the cells by pipetting, breaking up contacts
-Incubate the plate for 5 minutes at 37 °C and 5% CO_2
-Add 5 µL 10x anti-CD107a-blocking antibody and resuspend the cells by pipetting
-Incubate the plate for 5 minutes at 37 °C and 5% CO_2
-Wash by adding 100 µL of assay medium and centrifugate for 5 minutes at 500 g
-Remove supernatant
-Resuspend in 50 µL assay medium and mix cells by pipetting
-Spin down for 10 seconds to enable direct contact formation
-Incubate the plate for 10 minutes at 37 °C and 5% CO_2
-Add 5 µL 10x anti-CD107a-FITC and resuspend the cells by pipetting, breaking up contacts
-Incubate the plate for 5 minutes at 37 °C and 5% CO_2
-Add 5 µL 10x anti-CD107a-blocking antibody and resuspend the cells by pipetting
-Incubate the plate for 5 minutes at 37 °C and 5% CO_2
-Wash by adding 100 µL of assay medium and centrifugate for 5 minutes at 500 g

-Remove supernatant
-Resuspend in 50 µL assay medium and mix cells by pipetting
-Spin down for 10 seconds to enable direct contact formation
-Incubate the plate for 10 minutes at 37 °C and 5% CO_2
-Add 5 µL 10x anti-CD107a-APC and resuspend the cells by pipetting, breaking up contacts
-Incubate the plate for 5 minutes at 37 °C and 5% CO_2
-Wash by adding 100 µL of assay medium and centrifugate for 5 minutes at 500 g
-Remove supernatant
-Add 25 µL PBS + Zombie NIR 1:700 and resuspend the cells by pipetting
-Incubate the plate on ice for 10 minutes
-Add 25µL master mix and resuspend the cells by pipetting
-Incubate the plate on ice for 15 minutes
-Wash by adding 100 µL of PBS + 2 % FCS and centrifugate for 5 minutes at 500 g
-Remove supernatant
-Resuspend cells rigorously in 25 µL Enzyme Free Cell Dissociation Solution
-Incubate the plate on ice for 10 minutes
-Pipet again to remove cell-cell contacts
-Add 25 µL 4 % PFA and mix by pipetting
-Incubate the plate on ice for 10 minutes
-Wash by adding 100 µL of PBS + 2 % FCS and centrifugate for 5 minutes at 500 g
-Resuspend cells in desired volume for flow cytometry

Flow cytometry and data analysis

Flow cytometry was performed adhering to Guidelines for the use of flow cytometry and cell sorting in immunological studies (third edition) (Cossarizza, A.; Chang, H. D.; Radbruch, A.; Abrignani, S.; Addo, R.; Akdis, M.; Andrä, I.; et al., Eur. J. Immunol. 2021. 51: 2708–3145), using a Cytek® Aurora spectral flow cytometer. Analysis was performed with FlowJo version 10.10. General gating for NK cells is shown in supplementary figure 3. Data cleanup with FlowAI_v2.3.2 was performed on the data of the 3 repeats of 5 donors resulting in the same downstream result as without, so this step was left out for the rest. Further data analysis, statistic and visualization were performed with GraphPad Prism 10.3.1

Table of Antibodies and Reagents

Table 1: Table of Antibodies

Antibody (clone)	Conjugate	Supplier	Final-dilution / µg/mL
CD107a (H4A3)	BV421	BioLegend (San Diego)	1
CD107a (H4A3)	APC	BioLegend (San Diego)	1
CD107a (H4A3)	FITC	BioLegend (San Diego)	1
CD107a (H4A3)	unconjugated	BioLegend (San Diego)	10
CD16 (3G8)	BUV615	BD Bioscience (San Jose)	0.5
CD3 (UCHT1)	BUV563	BD Bioscience (San Jose)	1
CD56 (B159)	BUV805	BD Bioscience (San Jose)	0.4
CD69 (FN50)	BV750	BioLegend (San Diego)	0.5
CD56 (MEM-188)	PE	BioLegend (San Diego)	1

Table 2: Table of Reagents

Reagent	Supplier
DMEM	Thermo Fischer Scientific (Waltham)

DPBS	Thermo Fischer Scientific (Waltham)
Enzyme Free Cell Dissociation Solution	Merck KGaA, (Darmstadt)
Fetal calf serum (FCS)	Thermo Fischer Scientific (Waltham)
HEPES	Thermo Fischer Scientific (Waltham)
IMDM	Thermo Fischer Scientific (Waltham)
Paraformaldehyde (PFA)	Sigma-Aldrich (St. Louis)
Penicilin-Streptomycin (P/S)	Thermo Fischer Scientific (Waltham)
Recombinant human IL-15	PAN-Biotech (Aidenbach)
RPMI	Thermo Fischer Scientific (Waltham)
TrypLE^{TM} Express^{TM} (Trypsin)	Thermo Fischer Scientific (Waltham)
Zombie NIR	BioLegend (San Diego)

Supplementary Figures

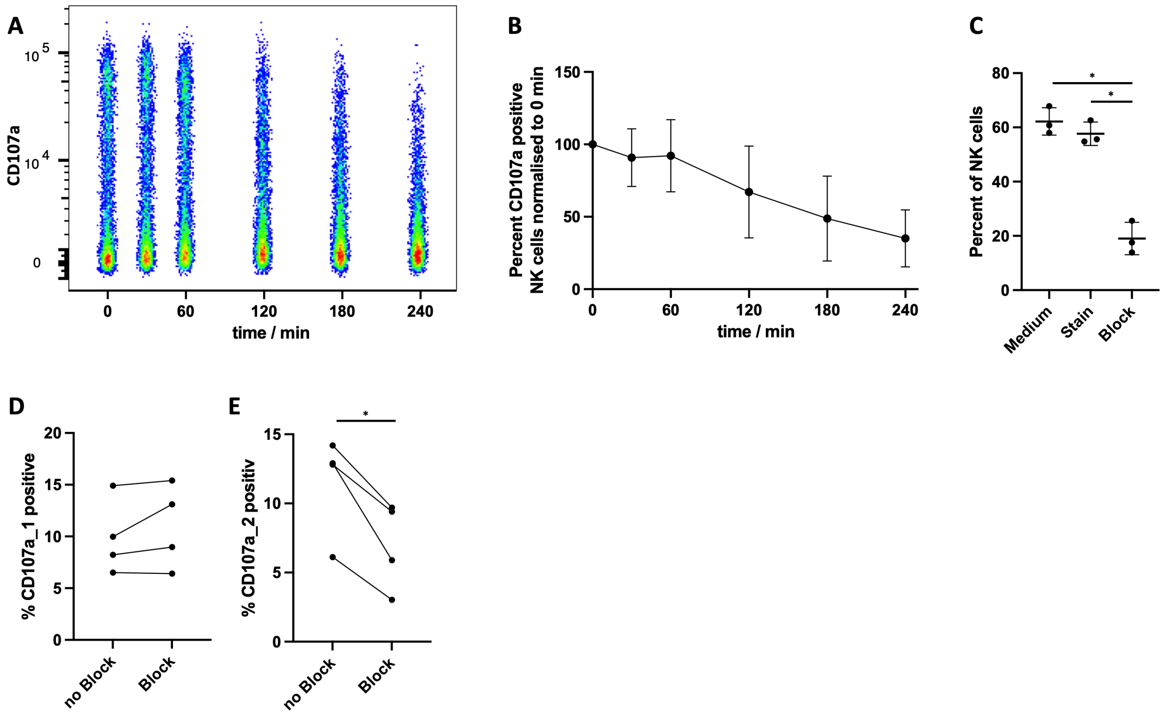

Figure S1: CD107a surface retention and blocking.

(A, B) Preactivated NK cells and adherent HeLa-CD48 target cells (E:T, 1:1) were co-incubated for 60 minutes, after which NK cells were separated from the adherent target cells. CD107a was stained on the NK cells at the indicated time points after separation. Exemplary plots (A) and percentage of CD107a positive NK cells normalized to t = 0 min (B). (C) K562 cells and preactivated NK cells (E:T, 1:2) were co-incubated for 40 minutes. Cells were then stained using a BV421 conjugated anti-CD107a antibody (stain), 10-fold unconjugated anti-CD107a antibody (block) or incubated in medium alone (medium). Subsequently, cells were washed and stained using a FITC conjugated anti-CD107a antibody. Percentage of FITC-CD107a positive NK cells is shown. (D, E) Effect of the blocking step on the first CD107a staining step (D) and the second CD107a staining step (E) during the multiple degranulation assay performed with PBMCs and K562 (E:T, 1:10). B: Mean and SD. n = 3, C: Repeated measures one way ANOVA with multiple comparison. *p ≤ 0.05. n = 3. E: paired t-test. *p ≤ 0.05. n = 4.

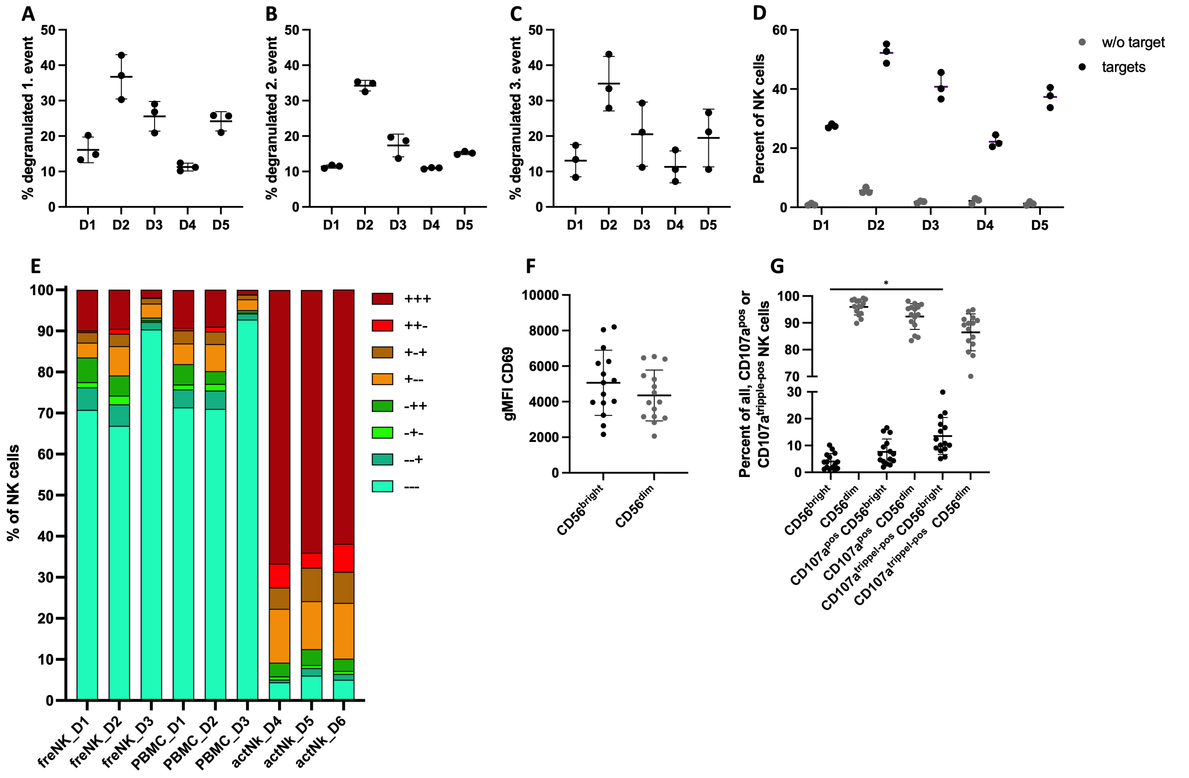

Supplementary Figure 2: Interexperimental variability, effect of NK cell isolation and pre-activation and degranulation of CD56^{dim} and CD56^{bright} NK cells.

(A, B, C, D, F, G) Data from multiple degranulation assay with PBMCs and K562 cells shown in Figure 1 C. (A, B, C) Percentage of NK cells positive for the first CD107a stain (A), the second CD107a stain (B) or the third CD107a stain (C) using PBMCs from five different donors (D1-D5) repeated three independent times. (D) Percentage of NK cells that did degranulate during the multiple degranulation assay with and without target cells present. (E) Degranulation outcomes using freshly isolated NK cells (freNK), the corresponding PBMCs or pre-activated NK cells (actNK). (F) Geometric mean fluorescence intensity of CD69 on CD56 bright and CD56 dim NK cells after the multiple degranulation assay. (G) Percentage of CD56^{bright} and CD56^{dim} NK cells among all NK cells, CD107a positive NK cells and CD107a triple positive NK cells. A – D, F, G: 3 repeats of 5 different donors as in Figure 1C, Mean and SD. F: Paired t-test p=0.054. G: Nested 1 way ANOVA with multiple comparison *$p \leq 0.05$.

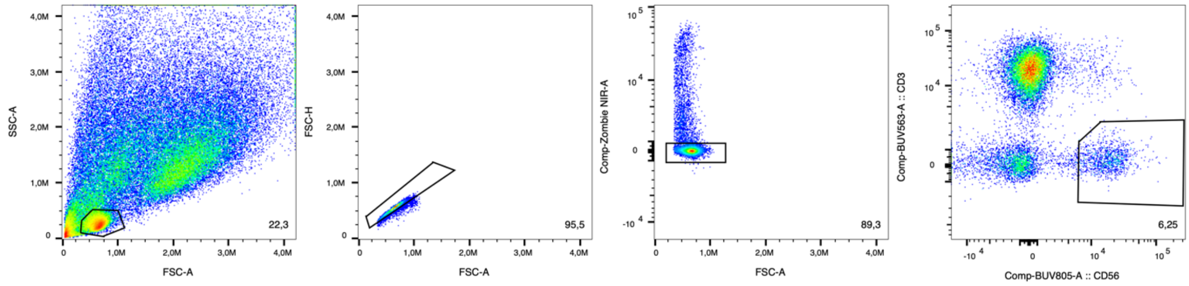

Supplementary Figure 3: Exemplary NK cell gating strategy.
NK cells were gated according to size, as single cells, negative for the dead cell dye Zombie NIR, CD3 negative and CD56 positive. Example taken from experiment with PBMCs and K662 cells.
